# Anatomic parameters for diagnosing congenital cervical stenosis via computed tomography

**DOI:** 10.1007/s00276-025-03797-4

**Published:** 2026-01-05

**Authors:** David Shin, Brandon Shin, Daniel Im, Timothy Tang, Chandler Dinh, Carson Cummings, Zachary Brandt, Kai Nguyen, Davis Carter, Mei Carter, Jacob Razzouk, Taha M. Taka, Gideon Harianja, Vahe Yacoubian, Muhammad Abd-El-Barr, Nathaniel Wycliffe, Wayne Cheng, Olumide Danisa

**Affiliations:** 1 Loma Linda University School of Medicine, Loma Linda, CA USA; 2 Department of Surgery, University of California Davis Medical Center, Sacramento, CA USA; 3 Department of Orthopaedic Surgery, Loma Linda University Medical Center, Loma Linda, CA USA; 4 Departments of Orthopaedic and Neurological Surgery, Duke University Healthcare Center, Durham, NC USA; 5 Department of Radiology, Loma Linda University Medical Center, Loma Linda, CA USA; 6 Division of Orthopaedic Surgery, Jerry L. Pettis Memorial Veterans Hospital, Loma Linda, CA USA; 7 Division of Spine Surgery, Duke University Health System (DUHS), 200 Trent Drive, Room 5330, Durham, NC 27710-04000 USA

**Keywords:** Cervical, Computed tomography, Stenosis, Neurology

## Abstract

**Purpose:**

To establish parameters for congenital cervical stenosis (CCS) using computed tomography (CT), assessing influences of patient sex, race, and ethnicity.

**Methods:**

Measurements were collected of anteroposterior diameter (APD), interpedicular distance (IPD) and cervical intervertebral foramen dimensions (CIFD) from 1000 patients between 18 and 35 years of age without spinal pathology. CCS was determined as two standard deviations below the mean of the collected measurements.

**Results:**

Irrespective of vertebral level, mean anatomic APD, CIFD and IPD measurements were as follows: 14.94 ± 1.99 mm for APD, 6.58 ± 1.45 mm and 6.68 ± 1.45 mm for left and right widths, of 9.30 ± 2.30 mm and 9.25 ± 2.80 mm for left and right heights, 57.0 ± 19.2 mm^2^ and 59.5 ± 20.3 mm^2^ for left and right areas, and 25.4 ± 1.78 mm for IPD. Irrespective of vertebral level, threshold values for CCS were 10.96 mm for APD, 3.68 mm and 3.78 mm for left and right widths, of 4.70 mm and 3.65 mm for left and right heights, 20.6 mm^2^ and 19 mm^2^ for left and right areas, and 21.8 mm for IPD. Males demonstrated larger CCS threshold values than females for left and right CIFD area and APD. African American patients had smaller CIFDs and APD, and subsequent CCS thresholds compared to White patients.

**Conclusions:**

This study reports measurements of CIFD, IPD, and APD to establish quantitative thresholds for diagnosis of CCS. CCS thresholds were significantly influenced by patient sex, race, and ethnicity.

**Supplementary Information:**

The online version contains supplementary material available at 10.1007/s00276-025-03797-4.

## Introduction

Congenital cervical stenosis (CCS) refers to a condition in which an individual has developmental narrowing of their cervical spinal canal [1]. CCS has been reported to cause symptoms of degenerative disease such as pain and cervical myelopathy, and proper diagnosis is essential for treatment and preventative interventions [2]. Current diagnosis often relies on interpretation of radiographic images, and the establishment of universally accepted diagnostic parameters of CCS has been a topic of discussion [1]. In a cadaveric study, Bajwa et al. utilized anatomical measurements of sagittal canal diameter (SCD), interpedicular distance (IPD), and pedicle length to determine parameters of diagnosing CCS [3]. IPD is often measured via radiographic imaging and has been shown to be an indirect measurement of vertebral foramen area [4]. Additional measurements often taken via radiographic imaging is that of anteroposterior diameter (APD) and cervical intervertebral foramen dimensions (CIFD) [5]. APD is clinically important in traumatic, degenerative, and inflammatory conditions, and congenital narrowing of the spinal canal has been associated with a greater risk of developing cervical spinal stenosis [6]. CIFD have been shown to be relevant in numerous spine surgical procedures such as correction of deformity and diagnosis of spinal pathology [7]. Additionally, cervical neuroforaminal stenosis has been shown to cause pain and worse outcomes in treatment of spinal stenosis [8]. Literature has investigated what constitutes normative and pathological measurements of spinal anatomy [5, 9, 10]. However, the relationship between spinal anatomy and diagnostic criteria of CCS remains unclear, and more objective, quantitative establishments of the parameters of CCS are needed. The aim of this study was to utilize measurements of APD, IPD, and CIFD via computed tomography (CT) to build upon the foundations established by current literature, as well as determine anatomic parameters of diagnosing CCS. Another goal of this study was to ​​evaluate for anatomical similarities or differences based on patient sex, race, and ethnicity.

## Materials and methods

### Patient selection

Following IRB approval (#5240130), we reviewed CT (GE Discovery 750 HD 64-slice CT scanner) scans of 1000 patients between 18 and 35 years of age free of spinal pathology. CT scans were of the cervical spine or soft tissue neck without contrast and were taken from January 2014 to January 2023. Patient consent was not required due to the nature of this retrospective, radiographic study. This study utilized similar methodology as established by Razzouk et al. in their morphometric analysis of CIFD [5]. Patients were included as long as they did not possess any spinal pathology that would exclude them from this study’s purpose and were excluded following the same criteria as Razzouk et al. which consisted of a history of disc degeneration, scoliosis with a measured coronal deformity greater than 10 degrees, spondylolisthesis, traumatic spinal injury with and without bone injury, infection, malignancy, neck, back, or upper extremity pain or numbness, existing spinal hardware, or previous spinal surgery. Scans were reviewed in a systematic, consecutive manner corresponding to when their imaging was completed. Relevant spinal pathology was identified based on previous diagnosis in either the patients radiographic or electronic medical records. Demographic information was recorded at time of imaging and were obtained via the patient’s self-reported answers in the electronic medical record.

## Data collection

**Fig. 1:**
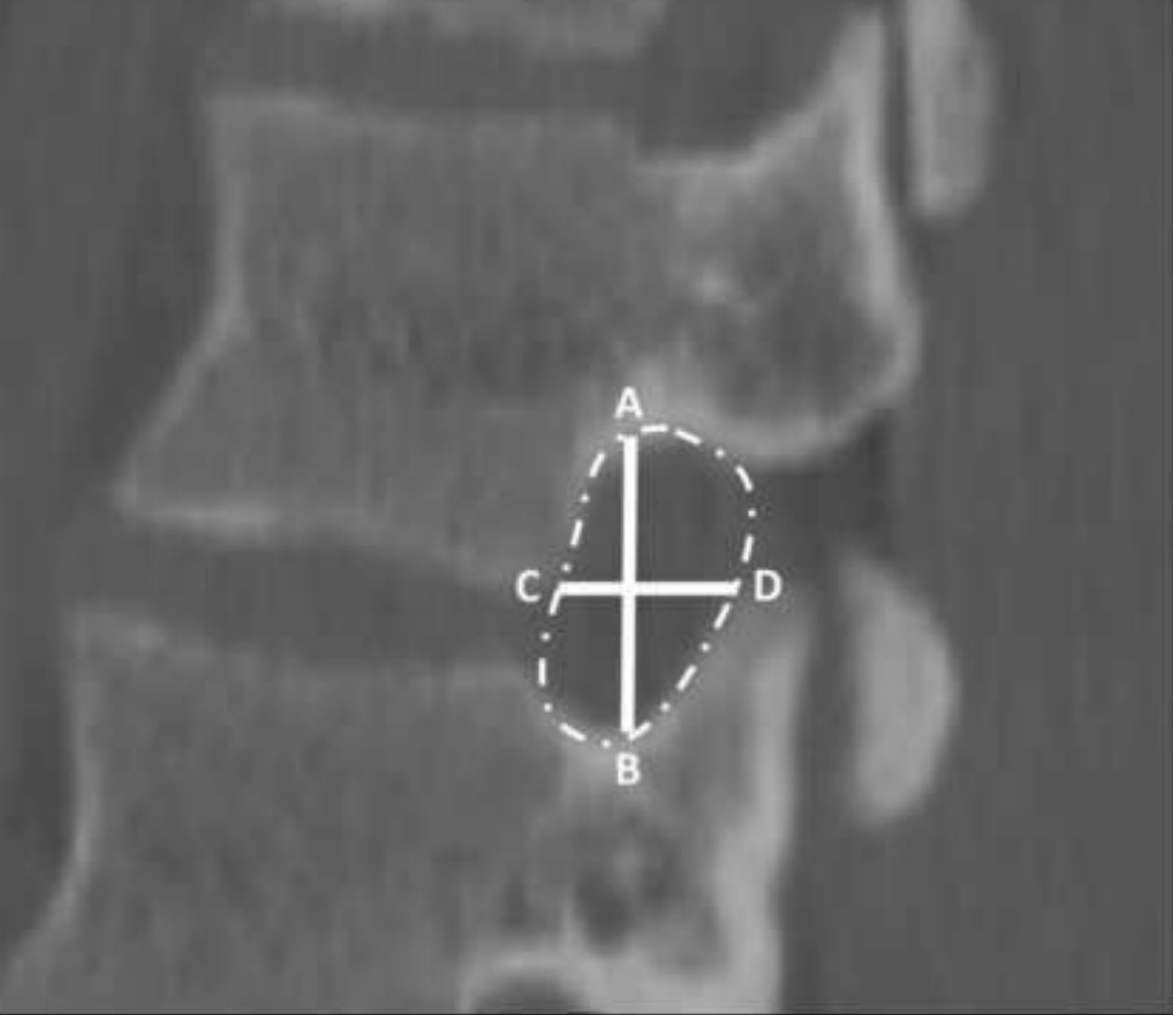
Cervical neuroforamina measurements. NFH was measured as illustrated by line *AB*. Sagittal AP width was measured as illustrated by line *CD*. Axial AP width was measured with the same methodology, though in the axial view. Foraminal area was measured as illustrated by the foramen contained within the hatched line

**Fig. 2:**
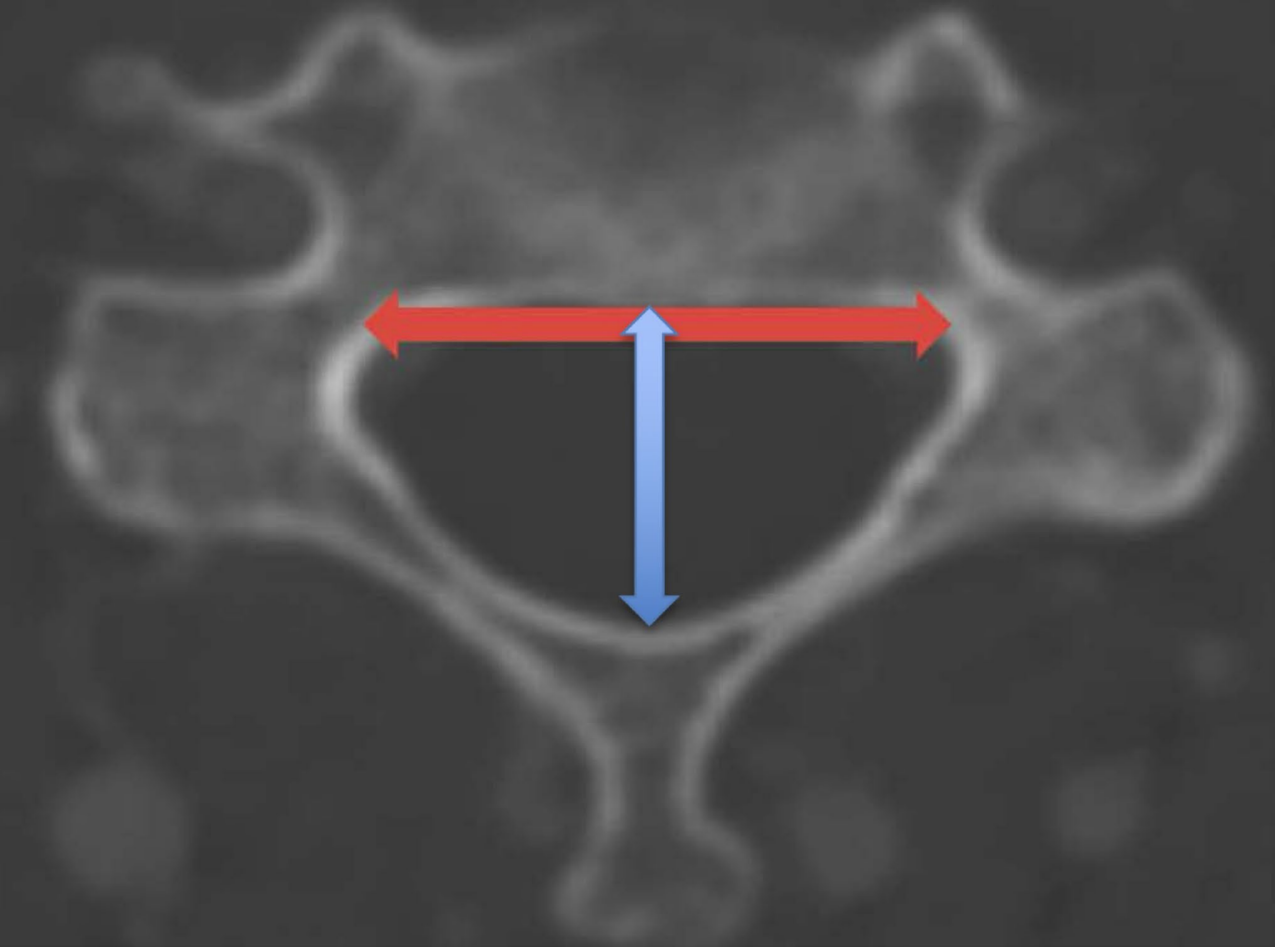
Measurement technique of cervical interpedicular distance and anteroposterior distance. IPD was measured as illustrated by the horizontal line, while APD was measured as illustrated by the vertical line

Similar to Razzouk et al. images were reviewed and measured using the IMPAX6 (Agfa-Gavaert, Mortsel, Belgium) picture archiving and communication system with window and level designations of 2000 Hounsfield Unit (HU) and 500 HU, respectively [5]. All measurements were performed by medical students trained by a board-certified neuroradiologist (NW) to measure APD, IPD, and CIFD, defined as follows: foraminal height, axial width, and area. Following the methods regarding CIFD detailed by Razzouk et al. the neuroforaminal area was calculated according to the borders of the foraminal bony outline using IMPAX6 tracing freeform tool in the sagittal view. Foraminal height was defined as the longest distance between the borders of the upper and lower pedicle viewed in the sagittal view. Neuroforaminal width was defined as the shortest distance between the posterior-inferior corner of the superior vertebra and the anterior border of the superior articular process of the lower vertebra as measured on the axial view. Coronal, sagittal, and axial views were utilized as visual aids, though measurements of width, height, and area were performed using solely the axial or sagittal plane [5]. APD was defined as the distance between the anterior surface of the spinous process and the posterior surface of the vertebral body. IPD was defined as the maximum distance between the medial aspect of the pedicles at a given vertebral level. Figure 1 provides an illustration of the measurement technique for intervertebral foramen dimensions. Figure 2 provides an illustration of the measurement technique for IPD and AP diameter. Accounting for the oblique trajectory of the foramina is important in CT when viewing straight sagittal cuts, but per Razzouk et al. oblique sagittal views were not utilized and axial measurements were employed to account for foraminal orientation as axial-derived and straight sagittal-derived measurements of foraminal width were found to be similar and did not possess clinically significant differences [5, 11].

Interobserver reliability utilized the intraclass correlation coefficient (ICC) two-way mixed model on absolute agreement [12, 13]. ICC was defined as poor, fair, good, or excellent based on threshold values of < 0.40, 0.40 to 0.59, 0.60 to 0.74, and > 0.75, respectively [12, 13]. For the first 200 subjects, each CT scan was measured by 2 reviewers to assess ICC, and ICC was determined to be excellent at 0.792 with a 95% confidence interval of 0.736 to 0.842. The remaining measurements were allowed to be performed by 1 reviewer per CT scan. Racial and ethnic categories were defined based on the Office of Management and Budget and National Institute of Health (NIH) National Institute on Minority Health and Health Disparities parameters [14]. CCS was determined as described by Bajwa et al. whereby values two standard deviations below the mean of the measurements conducted in this study were defined as congenitally stenotic [3].

## Statistical and power analyses

Data collection and visualization was performed using Microsoft Excel version 16.58 (Microsoft Corporation, 2022, Redmond, WA, USA) and statistical analyses were performed via SPSS version 28 (IBM Corporation, 2021, Armonk, NY, USA), with alpha defined as *p* < 0.05. Paired sample *t*-tests were also used to assess for differences between left- versus right-sided neuroforaminal measurements. Differences in APD, IPD, and CIFD based on sex, race, and ethnicity were assessed using two-way analysis of covariance (ANCOVA) with anthropometric covariates, estimated marginal means, and type III sum-of-square specification. Measurement differences among disc levels were analyzed using one-way analysis of variance (ANOVA) with *post-hoc* Bonferroni and Tukey corrections. Further analysis included Kolmogorov-Smirnov tests and Q-Q plots to assess data normality, Levene’s homogeneity of variance test and regression residual plots to evaluate homoscedasticity, and relationships between radiographic and demographic variables were assessed utilizing Pearson correlation tests [15, 16]. Power analyses for nondirectional partial correlation and one-way ANOVA were conducted using the parameters established by Razzouk et al. yielding an achieved power of 100% in both analyses [5].

## Results

### Cohort description

This study screened a total of 4762 subjects, of which 3762 were excluded. Of those excluded, 1309 were not between 18 and 35 years of age, 967 possessed incomplete or poor-quality imaging, 486 had history of previous spinal surgery, 354 had diagnosis of neck or upper extremity pain or numbness, 327 had CCS, 264 had traumatic spinal injury, 27 had spinal infection, 22 had malignancy, 4 had cerebral palsy, and 2 had achondroplasia. Patients with CCS were excluded as this study was intended to establish baseline anatomic parameters of CCS utilizing a “normal” patient population which was comprised of patients from 18 to 35 without pathology. Of the 1,000 patients included in this study, 486 were female and 514 were male. With respect to race and ethnicity, 357 were Hispanic, 245 were White, 144 were Black, 83 were Asian, 18 were identified as “other,” and for 153 patients racial and ethnic information was unavailable. Mean age was 25.9 ± 5.8 years, with a range of 18–35 years. Mean height was 1.68 ± 0.11 m, with a range of 1.30–1.92 m. Mean weight was 81.88 ± 24.11 kg (kg), with a range of 38.10 to 192.80 kg. Mean patient BMI was 27.68 ± 7.89 kg/m^2^, with a range of 15.62–68.17 kg/m^2^.

### CCS threshold measurements

Irrespective of vertebral level, mean anatomic CIFD, IPD, and APD measurements were as follows: 6.58 ± 1.45 mm and 6.68 ± 1.45 mm for left and right CIFD widths, of 9.30 ± 2.30 mm and 9.25 ± 2.80 mm for left and right CIFD heights, 57.0 ± 19.2 mm^2^ and 59.5 ± 20.3 mm^2^ for left and right CIFD areas, 25.4 ± 1.78 mm for IPD, and 14.94 ± 1.99 mm for APD. Irrespective of vertebral level, threshold values for CCS were 3.68 mm and 3.78 mm for left and right CIFD widths, of 4.70 mm and 3.65 mm for left and right CIFD heights, 20.6 mm^2^ and 19 mm^2^ for left and right CIFD areas, 21.8 mm for IPD, and 10.96 mm for APD. CIFD did not vary based on laterality. Tables 1, 2, 3, 4, 5 and 6 report CCS threshold values for anatomic measurements of CIFD, IPD, and AP diameter for each level C2-T1. Supplementary Table 1 reports mean anatomic measurements based on vertebral level. Supplementary Table 2 reports differences in anatomic measurements based on vertebral level.

**Table 1:** Measurement thresholds for CCS at C2–C3

Anatomic measurement	Total (*n* = 1000)		Male (*n* = 489)		Female (*n* = 511)		Male: Female
	Mean	Threshold for CCS	Mean	Threshold for CCS	Mean	Threshold for CCS	*p*
Left CIFD Width	6.9 ± 1.6	3.7	6.9 ± 1.6	3.7	7.2 ± 1.6	4.0	0.021
Left CIFD Height	9.4 ± 2.7	4.0	10.1 ± 3.2	3.7	8.8 ± 1.7	5.4	< 0.001
Left CIFD Area	65 ± 23.6	17.8	72.3 ± 23.7	24.9	57.8 ± 19.7	18.4	< 0.001
Right CIFD Width	7.1 ± 1.6	3.9	6.9 ± 1.7	3.5	7.3 ± 1.5	4.3	0.010
Right CIFD Height	9.4 ± 3.7	2.0	10.1 ± 4.4	1.3	8.9 ± 1.7	5.5	< 0.001
Right CIFD Area	64.6 ± 23.8	17.0	72.1 ± 24.2	23.7	58.3 ± 20.5	17.3	< 0.001
IPD C3	24.2 ± 1.5	21.2	24.4 ± 1.7	21.0	23.8 ± 1.2	21.4	< 0.001
APD	15.5 ± 2.2	11.1	15.6 ± 2.2	11.2	15.4 ± 2.2	11.0	< 0.001

**Table 2:** Measurement thresholds for CCS at C3–C4

Anatomic measurement	Total (*n* = 1000)		Male (*n* = 489)		Female (*n* = 511)		Male: Female
	Mean	Threshold for CCS	Mean	Threshold for CCS	Mean	Threshold for CCS	*p*
Left CIFD Width	6.4 ± 1.6	3.2	6.2 ± 1.6	3.0	6.6 ± 1.5	3.6	0.003
Left CIFD Height	8.6 ± 2.4	3.8	9.1 ± 2.9	3.3	8.3 ± 1.5	5.3	< 0.001
Left CIFD Area	54.7 ± 18.4	17.9	59.1 ± 18.9	21.3	51.3 ± 16.2	18.9	< 0.001
Right CIFD Width	6.3 ± 1.5	3.3	6.1 ± 1.6	2.9	6.6 ± 1.5	3.6	< 0.001
Right CIFD Height	8.5 ± 1.6	5.3	9.1 ± 1.7	5.7	8.3 ± 1.4	5.5	< 0.001
Right CIFD Area	55.1 ± 19.6	15.9	60.3 ± 19.6	21.1	51.4 ± 17.5	16.4	< 0.001
IPD C4	25.2 ± 1.7	21.8	25.4 ± 1.7	22.0	24.7 ± 1.5	21.7	< 0.001
APD	14.1 ± 1.7	10.7	14.3 ± 1.7	10.9	14.0 ± 1.7	10.6	< 0.001

**Table 3:** Measurement thresholds for CCS at C4–C5

Anatomic measurement	Total (*n* = 1000)		Male (*n* = 489)		Female (*n* = 511)		Male: Female
	Mean	Threshold for CCS	Mean	Threshold for CCS	Mean	Threshold for CCS	*p*
Left CIFD Width	6.5 ± 1.4	3.7	6.4 ± 1.4	3.6	6.7 ± 1.5	3.7	0.006
Left CIFD Height	9 ± 1.6	5.8	9.4 ± 1.8	5.8	8.8 ± 1.6	5.6	< 0.001
Left CIFD Area	57.9 ± 19	19.9	62.9 ± 19.5	23.9	53.9 ± 16.8	20.3	< 0.001
Right CIFD Width	6.5 ± 1.4	3.7	6.4 ± 1.4	3.6	6.6 ± 1.4	3.8	0.012
Right CIFD Height	8.8 ± 1.6	5.6	9.3 ± 1.7	5.9	8.8 ± 4.4	0.0	0.070
Right CIFD Area	58.2 ± 19.5	19.2	63.8 ± 20.2	23.4	53.8 ± 17	19.8	< 0.001
IPD C5	25.8 ± 1.9	22.0	26.1 ± 1.9	22.3	25.2 ± 1.6	22.0	< 0.001
APD	14.3 ± 1.6	11.1	14.5 ± 1.6	11.3	14.1 ± 1.6	10.9	< 0.001

**Table 4:** Measurement thresholds for CCS at C5–C6

Anatomic measurement	Total (*n* = 1,000)		Male (*n* = 489)		Female (*n* = 511)		Male: Female
	Mean	Threshold for CCS	Mean	Threshold for CCS	Mean	Threshold for CCS	*p*
Left CIFD Width	6.4 ± 1.4	3.6	6.3 ± 1.5	3.3	6.6 ± 1.3	4.0	0.003
Left CIFD Height	9.5 ± 3.3	2.9	9.9 ± 4.1	1.7	9.1 ± 1.6	5.9	0.002
Left CIFD Area	59.9 ± 18.3	23.3	63.8 ± 19.1	25.6	56.7 ± 16.5	23.7	< 0.001
Right CIFD Width	6.6 ± 1.4	3.8	6.3 ± 1.4	3.5	6.9 ± 1.4	4.1	< 0.001
Right CIFD Height	9.5 ± 5.1	-0.7	9.6 ± 1.9	5.8	9.5 ± 6.9	-4.3	0.790
Right CIFD Area	60.3 ± 19.4	21.5	64.5 ± 20.1	24.3	57.6 ± 18.8	20.0	< 0.001
IPD C6	26.2 ± 1.9	22.4	26.5 ± 1.9	22.7	25.7 ± 1.6	22.5	< 0.001
APD	14.7 ± 1.9	10.9	15.0 ± 1.9	11.2	14.3 ± 1.8	10.7	< 0.001

**Table 5:** Measurement thresholds for CCS at C6–C7

Anatomic measurement	Total (*n* = 1000)		Male (*n* = 489)		Female (*n* = 511)		Male: Female
	Mean	Threshold for CCS	Mean	Threshold for CCS	Mean	Threshold for CCS	*p*
Left CIFD Width	6.6 ± 1.3	4.0	6.5 ± 1.3	3.9	6.8 ± 1.5	3.8	0.024
Left CIFD Height	9.6 ± 1.8	6.0	10.0 ± 2.0	6.0	9.3 ± 1.8	5.7	< 0.001
Left CIFD Area	58.7 ± 17	24.7	62.5 ± 18.0	26.5	56.2 ± 16.5	23.2	< 0.001
Right CIFD Width	6.8 ± 1.4	4.0	6.6 ± 1.4	3.8	7.0 ± 1.5	4.0	< 0.001
Right CIFD Height	9.7 ± 2.9	3.9	10.0 ± 2.0	6.0	9.5 ± 3.7	2.1	0.033
Right CIFD Area	60.7 ± 20.3	20.1	65.4 ± 21.2	23.0	57.3 ± 19	19.3	< 0.001
IPD C7	25.4 ± 1.9	21.6	25.7 ± 1.9	21.9	25.0 ± 1.6	21.8	< 0.001
APD	15.3 ± 2.2	10.9	15.8 ± 2.2	11.4	14.9 ± 2.1	10.7	< 0.001

**Table 6:** Measurement thresholds for CCS at C7–T1

Anatomic Measurement	Total (*n* = 1000)		Male (*n* = 489)		Female (*n* = 511)		Male: Female
	Mean	Threshold for CCS	Mean	Threshold for CCS	Mean	Threshold for CCS	*p*
Left CIFD Width	6.7 ± 1.4	3.9	6.8 ± 1.4	4.0	6.6 ± 1.4	3.8	0.028
Left CIFD Height	9.7 ± 2	5.7	10.2 ± 2	6.2	9.2 ± 1.9	5.4	< 0.001
Left CIFD Area	57.7 ± 19	19.7	63.5 ± 19.6	24.3	52.9 ± 17.1	18.7	< 0.001
Right CIFD Width	6.8 ± 1.4	4.0	6.8 ± 1.4	4.0	6.7 ± 1.4	3.9	0.503
Right CIFD Height	9.6 ± 1.9	5.8	10.2 ± 2.0	6.2	9.1 ± 1.7	5.7	< 0.001
Right CIFD Area	58.2 ± 18.9	20.4	64.5 ± 19.9	24.7	52.9 ± 16.9	19.1	< 0.001
IPD	*	*	*	*	*	*	*
APD	16.1 ± 2.4	11.3	16.7 ± 2.3	12.1	15.6 ± 2.3	11.0	< 0.001

*Interpedicular distance (IPD) measurements at C7–T1 were unavailable because the analysis was limited to cervicalspine dimensions.

### Influence of sex, race, and ethnicity

A few significant differences based on patient sex were found but not consistently observed across all vertebral levels. Males demonstrated larger CCS threshold values compared to females for left and right CIFD area and APD at all vertebral levels, while females demonstrated a larger CCS threshold for left and right CIFD width at C2–C3, C3–C4, C4–C5, and C5–C6. While sex-based differences in left and right CIFD height and IPD thresholds were also observed, one sex was not consistently larger or smaller than the other across vertebral levels. Tables 1, 2, 3, 4, 5 and 6 report CCS threshold values based on patient sex from C2-T1. Supplementary Table 3 reports differences in CIFD, IPD, and APD measurements based on patient sex per vertebral level. Significant differences were observed based on patient race and ethnicity when analyzed per vertebral level from C2–T1, as well as when analyzed irrespective of vertebral level. Supplementary Table 4 reports mean CIFD, IPD, and APD measurements based on race and ethnicity per vertebral level. Supplementary Table 5 reports mean differences in measurement values amongst racial and ethnic groups per vertebral level. Table 7 reports CCS threshold values based on patient race and ethnicity per vertebral level. African American patients had significantly smaller APD measurements compared to white patients. Across all other anatomic variables, one racial group did not have consistently larger or smaller measurements. For instance, while Asian and white patients had larger left width compared to their Hispanic and African American counterparts, Hispanics tended to have larger right CIFD widths.

**Table 7:** Congenital cervical stenosis thresholds based on race and ethnicity

Anatomic measurement	Race/	C2–C3		C3–C4		C4–C5		C5–C6		C6–C7		C7–T1	
	Ethnicity	Mean	Threshold for CCS	Mean	Threshold for CCS	Mean	Threshold for CCS	Mean	Threshold for CCS	Mean	Threshold for CCS	Mean	Threshold for CCS
Left CIFD Width	Caucasian	7.1 ± 1.5	4.1	6.4 ± 1.5	3.4	6.6 ± 1.6	3.4	6.3 ± 1.4	3.5	6.6 ± 1.2	4.2	6.8 ± 1.4	4.0
	Hispanic	7 ± 1.5	4.0	6.4 ± 1.6	3.2	6.5 ± 1.4	3.7	6.5 ± 1.4	3.7	6.7 ± 1.4	3.9	6.7 ± 1.4	3.9
	African American	6.8 ± 2	2.8	6.3 ± 1.8	2.7	6.4 ± 1.6	3.2	6.4 ± 1.5	3.4	6.7 ± 1.7	3.3	6.5 ± 1.5	3.5
	Asian	7.1 ± 1.5	4.1	6.6 ± 1.3	4.0	6.5 ± 1.6	3.3	6.3 ± 0.8	4.7	6.2 ± 1.4	3.4	6.5 ± 1.5	3.5
Left CIFD Height	Caucasian	9.9 ± 1.9	6.1	9.1 ± 3.7	1.7	9.4 ± 1.8	5.8	9.6 ± 1.7	6.2	9.8 ± 1.7	6.4	10.2 ± 2	6.2
	Hispanic	9.5 ± 3.3	2.9	8.7 ± 1.7	5.3	9.1 ± 1.7	5.7	9.7 ± 4.1	1.5	9.8 ± 2	5.8	9.8 ± 2.1	5.6
	African American	9.3 ± 1.8	5.7	8.4 ± 1.7	5.0	8.7 ± 1.7	5.3	9 ± 1.6	5.8	9.3 ± 1.7	5.9	9.4 ± 1.8	5.8
	Asian	9.3 ± 1.8	5.7	8.7 ± 1.5	5.7	9 ± 1.9	5.2	9.2 ± 1.6	6.0	8.9 ± 1.8	5.3	9.1 ± 1.9	5.3
Left CIFD Area	Caucasian	70.5 ± 21.8	26.9	58.7 ± 16.5	25.7	61.8 ± 18.9	24	63.4 ± 17.9	27.6	63.2 ± 16.8	29.6	64.1 ± 19.4	25.3
	Hispanic	64.9 ± 24.1	16.7	55.9 ± 19.1	17.7	59.1 ± 18.9	21.3	61 ± 19	23.0	59.9 ± 18	23.9	58.4 ± 19	20.4
	African American	63.1 ± 20.6	21.9	51.5 ± 16.1	19.3	54.5 ± 17.9	18.7	57.3 ± 15.6	26.1	56.2 ± 15.4	25.4	53.1 ± 16.4	20.3
	Asian	62.6 ± 25.5	11.6	54.2 ± 18.4	17.4	53.4 ± 18.6	16.2	50.9 ± 14.2	22.5	50.8 ± 15.1	20.6	52.1 ± 20	12.1
Right CIFD Width	Caucasian	7.1 ± 1.5	4.1	6.3 ± 1.6	3.1	6.4 ± 1.4	3.6	6.4 ± 1.4	3.6	6.6 ± 1.3	4.0	6.8 ± 1.5	3.8
	Hispanic	7.1 ± 1.6	3.9	6.4 ± 1.5	3.4	6.5 ± 1.4	3.7	6.6 ± 1.4	3.8	6.8 ± 1.4	4.0	6.8 ± 1.3	4.2
	African American	6.8 ± 1.6	3.6	6.2 ± 1.6	3.0	6.3 ± 1.4	3.5	6.7 ± 1.6	3.5	6.8 ± 1.7	3.4	6.7 ± 1.6	3.5
	Asian	7.4 ± 1.9	3.6	6.3 ± 1.7	2.9	6.7 ± 1.5	3.7	7 ± 1.5	4.0	6.9 ± 1.8	3.3	6.5 ± 1.1	4.3
Right CIFD Height	Caucasian	9.8 ± 1.9	6	8.9 ± 1.6	5.7	9.6 ± 5.8	− 2	9.9 ± 6.2	− 2.5	10 ± 1.9	6.2	9.9 ± 1.9	6.1
	Hispanic	9.6 ± 4.5	0.6	8.7 ± 1.7	5.3	9 ± 1.7	5.6	9.4 ± 2	5.4	9.9 ± 3.5	2.9	9.8 ± 2	5.8
	African American	9.2 ± 1.9	5.4	8.6 ± 1.7	5.2	8.7 ± 1.5	5.7	9.9 ± 8.9	− 7.9	9.4 ± 1.9	5.6	9.3 ± 1.4	6.5
	Asian	9.3 ± 2.1	5.1	8.7 ± 1.5	5.7	8.6 ± 1.7	5.2	9.1 ± 2.1	4.9	9.4 ± 2.1	5.2	9.4 ± 2.2	5.0
Right CIFD Area	Caucasian	70.7 ± 21.9	26.9	59.4 ± 18.4	22.6	62.8 ± 18.3	26.2	64.4 ± 18.6	27.2	65.8 ± 20.7	24.4	62.8 ± 19.2	24.4
	Hispanic	65.8 ± 24.1	17.6	56.9 ± 19.5	17.9	59.2 ± 20	19.2	61.3 ± 20.3	20.7	61.7 ± 20.8	20.1	59.6 ± 19.9	19.8
	African American	61.1 ± 20.9	19.3	52.3 ± 17.2	17.9	57 ± 17.3	22.4	59.2 ± 16.8	25.6	58.3 ± 16.7	24.9	54.6 ± 15.7	23.2
	Asian	59.1 ± 27.6	3.9	52.5 ± 23.7	5.1	54.3 ± 27.6	− 0.9	56.5 ± 31.4	− 6.3	56.3 ± 31.3	− 6.3	57.5 ± 28.1	1.3
IPD	Caucasian	24.6 ± 1.7	21.2	25.5 ± 1.8	21.9	26.2 ± 2	22.2	26.6 ± 1.8	23.0	25.8 ± 1.9	22.0	***	***
	Hispanic	24 ± 1.5	21.0	25 ± 1.6	21.8	25.6 ± 1.8	22.0	1.9 ± 0.9	0.1	25.2 ± 1.8	21.6	***	***
	African American	24 ± 1.6	20.8	25 ± 1.4	22.2	25.7 ± 1.7	22.3	26.1 ± 1.8	22.5	25.3 ± 1.8	21.7	***	***
	Asian	24.2 ± 1.6	21.0	24.8 ± 1.8	21.2	25.7 ± 1.7	22.3	26.3 ± 2.6	21.1	25.4 ± 1.9	21.6	***	***
APD	Caucasian	15.4 ± 2.1	11.2	14.3 ± 1.8	10.7	14.5 ± 1.7	11.1	14.9 ± 1.9	11.1	15.6 ± 2.4	10.8	16.4 ± 2.4	11.6
	Hispanic	15.2 ± 2.3	10.6	14.1 ± 1.6	10.9	14.2 ± 1.6	11.0	14.8 ± 1.8	11.2	15.3 ± 2.2	10.9	16.1 ± 2.4	11.3
	African American	14.6 ± 2.1	10.4	13.7 ± 1.8	10.1	13.9 ± 1.7	10.5	13.9 ± 2.2	9.5	14.5 ± 2.0	10.5	15.4 ± 2.1	11.2
	Asian	14.7 ± 2.2	10.3	13.8 ± 2.1	9.6	14.0 ± 2.0	10.0	14.1 ± 2.2	9.7	14.6 ± 2.3	10.0	15.6 ± 2.1	11.4

*Interpedicular distance (IPD) measurements at C7–T1 were unavailable because the analysis was limited to cervicalspine dimensions.

## Discussion

This study seeks to utilize CT to measure various anatomical distances of APD, IPD, and foraminal height, width, and area in the cervical spine in order to determine thresholds for the diagnosis of CCS, as well as to compare demographic based differences in threshold measurements for CCS. The use of CT diverges from other investigations which have purported MRI as the standard for measurement of cervical anatomic measurements, and subsequently, diagnosis of CCS [17]. Other literature also proposes that MRI classifications for congenital lumbar stenosis (CLS) can predict the risk for CCS [18]. However, prior investigations have demonstrated that CT scan measurements are also highly correlated with direct anatomic measurements [19, 20]. Thus, the use of CT for evaluation of cervical anatomic characteristics serves as an appropriate measurement tool.

In regard to threshold measurements, Bajwa et al. defined CCS at each level as a total canal area of: C3/4 = 1.82 cm^2^, C4/5 = 1.80 cm^2^, C5/6 = 1.84 cm^2^, C6/7 = 1.89 cm^2^, C7/T1 = 1.88 cm^2^. This definition relied on a calculation utilizing IPD: Total area of canal = area of rectangle (IPD × PL) + area of isosceles triangle {IPD × (SCD − PL)/2}. IPD lower limit values were as follows in mm: C3 = 22, C4 = 22.5, C5 = 22.5, C6 = 23, C7 = 22.5 [3]. Our investigation utilized IPD, bilateral CIFD height, area, and width, and analyzed them separately, rather than combining them into one score. Literature showcases investigation into analyzing anatomic parameters for diagnosing congenital stenosis, but only a few studies have done so via CIFD [21]. Ozaki et al. looked directly at measurements of neuroforaminal stenosis, but did not utilize their results to diagnose CCS, and did not set any thresholds [22]. Other researchers have utilized alternative anatomic measurements, such as “coronal vertebral body, AP vertebral body, pedicle width, pedicle length, laminar length, AP lateral mass, posterior canal distance, lamina-pedicle angle, and lamina-disc angle (LDA)” to define cervical stenosis [17]. Although these variables digress from the utilization of SCD, IPD, and pedicle length by Bajwa et al. our investigation sought to utilize novel variables alongside the previously utilized metric of IPD, in order to lend credibility to the use of CT measurement of APD and CIFD as a means for diagnosing CCS. Our CCS measurements for IPD were as follows: C3 = 21.2, C4 = 21.8, C5 = 22, C6 = 22.4, C7 = 21.6. These values were within 1 mm of the IPD thresholds of Bajwa et al. demonstrating a consistent determination of CCS amongst our population.

Regarding APD, our CCS threshold was 10.96 mm when considered irrespective of vertebral level. While there is no official consensus for APD in CCS, literature has showcased measurements of less than 12–13 mm to be indicative of CCS, in line with the results of our findings, and that an APD of less than 10 mm is associated with symptoms of myelopathy [1, 6, 23, 24]. As such, our baseline parameters for APD may help identify and manage patients who are at higher risk for developing myelopathy or radiculopathy, even before symptoms manifest. Current investigation also continues to emphasize the importance of early recognition and treatment of CCS. Bajwa et al. demonstrate an association between the presence of CCS, and lumbar and thoracic spinal stenosis. Thus, early recognition of the presence of CCS plays an important role in screening patients for the development of other neurologic deficits and abnormalities [25, 26]. Cervical stenosis, particularly when congenital, often remains asymptomatic until advanced age, strenuous physical activity or injury, and additional risk factors exacerbate its effects. Additionally, CCS typically presents with a more stable progression compared to the degenerative form [27]. While clinical diagnosis and surgical intervention of cervical stenosis involves multiple factors, especially symptomology, the establishment of baseline parameters through radiographic analysis can assist with timely detection and management of asymptomatic patients. Early interventions in diet, physical activity, and overall lifestyle could potentially prevent severe symptomatic complications and allow for clinicians to formulate a more tailored approach to patient management, including decisions regarding surgical intervention or conservative treatment.

### Influence of patient sex, race, and ethnicity on CCS thresholds

In line with other literature which proposes a predisposition for CCS in the black population compared to their white counterparts, this paper demonstrates that African Americans have smaller CIFDs and APD, and subsequent CCS thresholds at every vertebral level compared to their White counterparts [1]. Furthermore, our findings demonstrate that, irrespective of vertebral level, African Americans also have statistically significantly smaller left CIFD Width, but larger left CIFD Area compared to their Asian counterparts, and Hispanic patients have smaller left and right CIFD Area compared to their White counterparts. Asians also have smaller right CIFD Area compared to their white, Hispanic, and African American peers. Thus, while our results demonstrate significant differences between racial groups, they fail to demonstrate consistent race-based patterns across all vertebral levels or between anatomic characteristics. Our findings also demonstrate statistically significant differences between male and female patients, with men having significantly larger thresholds for APD and bilateral CIFD area compared to women across all vertebral levels. However, while differences in left and right CIFD height and IPD thresholds were also observed, they were not consistent across vertebral levels.

Given that males demonstrate a higher prevalence of cervical myelopathy, these larger threshold values may indicate a “lower threshold” for developing CCS compared to female counterparts [28]. There is sparse data regarding sex-specific comorbidities which place males at higher risk for stenosis. Some proposals include patient specific factors such as involvement in contact sports, weight-bearing occupations, and smoking [28, 29]. Further research should investigate the ways in which various social exposures can impact the greater incidences of myelopathy in men, in order to eliminate the presence of confounding factors which may lead to these relationships. Alternatively, investigators should consider the intersectionality between these sex and race-based differences in incidence to address the underlying disparities which differentially affect various communities across the United States.

### Limitations

Similarly, to previous studies, this study utilized a patient population of asymptomatic individuals from ages 18 to 35, excluding symptomatic and asymptomatic patients of ages below 18 and above 35. Thus, as aging and spinal pathology can compress the central canal and foramina, these measurements do not reflect the entire population of the United States [30]. While this was performed to establish baseline parameters, future studies can investigate vertebral measurements of CIFD, APD, and IPD of symptomatic patients to allow for the comparison of pathological measurements with the thresholds determined in this study, as well as determine normal anatomic parameters for growing and adolescent patients to determine proper growth and detect early manifestations of CCS.

Furthermore, with respect to race and ethnicity, our patient demographics were as follows: 357 were Hispanic, 245 were White, 144 were Black, 83 were Asian, 18 were identified as “other,” and for 153 patients racial and ethnic information was unavailable. While this is reflective of our institution’s patient population, it is not necessarily representative of larger demographics across the United States. This potentially limits the generalizability of our findings. Thus, it may be beneficial to engage in a larger national or international study to help develop more accurate ranges for “normal” measurements across different ethnic and gender groups.

Finally, another limitation is the absence of patient-reported outcome measures (PROMs) such as the Neck Disability Index (NDI) and Visual Analog Scale (VAS), which could provide clinical insight into the functional and symptomatic relevance of this study’s established anatomic parameters. While literature has showcased APD measurements of less than 12–13 mm to be indicative of CCS, in line with the results of our findings, many patients are often asymptomatic as symptoms of myelopathy are associated with an APD of less than 10 mm [1, 6, 23, 24]. Without PROMs, it is difficult to correlate our radiographic findings directly with the likelihood or severity of neurological symptoms associated with the identified anatomic parameters. While our radiographic analysis was intended to establish a baseline and possibly assist in identifying patients who are at higher risk for developing myelopathy or radiculopathy, future studies can incorporate outcome measures to enhance the clinical relevance of the established anatomic parameters and better inform patient management strategies.

## Conclusion

Irrespective of vertebral level, mean anatomic AP diameter, CIFD, and IPD measurements were as follows: 14.94 ± 1.99 mm for APD, 6.58 ± 1.45 mm and 6.68 ± 1.45 mm for left and right widths, of 9.30 ± 2.30 mm and 9.25 ± 2.80 mm for left and right heights, 57.0 ± 19.2 mm^2^ and 59.5 ± 20.3 mm^2^ for left and right areas, and 25.4 ± 1.78 mm for IPD. Irrespective of vertebral level, threshold values for CCS were 10.96 mm for AP diameter, 3.68 mm and 3.78 mm for left and right widths, of 4.70 mm and 3.65 mm for left and right heights, 20.6 mm^2^ and 19 mm^2^ for left and right areas, and 21.8 mm for IPD. This study aimed to contribute to establishing CCS parameters that may be used in conjunction with traditional radiographic analysis and symptomology in determining the presence of stenosis in patients with radiographically or clinically unclear presentation. These findings may help explain differences in predisposition or prevalence of cervical nerve root compression amongst patients of different races, which can be important when considering rates of surgery and access to care.

## Supplementary Information

Below is the link to the electronic supplementary material.

Supplementary Material 1

Supplementary Material 2

Supplementary Material 3

Supplementary Material 4

Supplementary Material 5

## Data Availability

The datasets used and/or analyzed during the current study are available from the corresponding author upon reasonable request.
