# Supplementary material for: Anatomic parameters for diagnosing congenital cervical stenosis via computed tomography
Source: Surg Radiol Anat. 2026 Jan 5;48(1):32. doi: 10.1007/s00276-025-03797-4 (PMC12769553; doi:10.1007/s00276-025-03797-4)
Supplement: Supplementary file 1 — Supplementary Material 1 [file 276_2025_3797_MOESM1_ESM.docx]

| **Supplementary Table 1. Mean CNFD, IPD, and APD** | | | | | | | | |
| --- | --- | --- | --- | --- | --- | --- | --- | --- |
| **Disc Level** | **Left NFD** | | | **Right NFD** | | | **IPD** | **APD** |
|  | **Width** | **Height** | **Area** | **Width** | **Height** | **Area** | **Mean (SD)** | **Mean (SD)** |
| *C2 - C3* | 6.9 ± 1.6 | 9.4 ± 2.7 | 65.0 ± 23.6 | 7.1 ± 1.6 | 9.4 ± 3.7 | 64.6 ± 23.8 | 24.2 ± 1.5 | 15.4 ± 2.3 |
| *C3 - C4* | 6.4 ± 1.6 | 8.6 ± 2.4 | 54.7 ± 18.4 | 6.3 ± 1.5 | 8.5 ± 1.6 | 55.1 ± 19.6 | 25.2 ± 1.7 | 14.1 ± 1.7 |
| *C4 - C5* | 6.5 ± 1.4 | 9.0 ± 1.6 | 57.9 ± 19.0 | 6.5 ± 1.4 | 8.8 ± 1.6 | 58.2 ± 19.5 | 25.8 ± 1.9 | 14.3 ± 1.6 |
| *C5 - C6* | 6.4 ± 1.4 | 9.5 ± 3.3 | 59.9 ± 18.3 | 6.6 ± 1.4 | 9.5 ± 5.1 | 60.3 ± 19.4 | 26.2 ± 1.9 | 14.7 ± 1.9 |
| *C6 - C7* | 6.6 ± 1.3 | 9.6 ± 1.8 | 58.7 ± 17.0 | 6.8 ± 1.4 | 9.7 ± 2.9 | 60.7 ± 20.3 | 25.4 ± 1.9 | 15.3 ± 2.2 |
| *C7 - T1* | 6.7 ± 1.4 | 9.7 ± 2.0 | 57.7 ± 19.0 | 6.8 ± 1.4 | 9.6 ± 1.9 | 58.2 ± 18.9 | * | 16.1 ± 2.4 |
