# Supplementary material for: Anatomic parameters for diagnosing congenital cervical stenosis via computed tomography
Source: Surg Radiol Anat. 2026 Jan 5;48(1):32. doi: 10.1007/s00276-025-03797-4 (PMC12769553; doi:10.1007/s00276-025-03797-4)
Supplement: Supplementary file 2 — Supplementary Material 2 [file 276_2025_3797_MOESM2_ESM.docx]

| **Supplementary Table 2. Differences in Cervical NFD, IPD, and APD Measurements based on Disc Level** | | | | | | | | | | | | | | | | | |
| --- | --- | --- | --- | --- | --- | --- | --- | --- | --- | --- | --- | --- | --- | --- | --- | --- | --- |
| **Level of Reference** | **Level of Comparison** | **Mean Difference (Reference - Comparison)** | | | | | | | | | | | | | | | |
|  |  | ***Left NFD Width*** | | ***Left NFD Height*** | | ***Left NFD Area*** | | ***Right NFD Width*** | | ***Right NFD Height*** | | ***Right NFD Area*** | | ***IPD*** | | ***APD*** | |
|  |  | **MD** | ***p*** | **MD** | ***p*** | **MD** | ***p*** | **MD** | ***p*** | **MD** | ***p*** | **MD** | ***p*** | **MD** | ***p*** | ***MD*** | ***p*** |
| *C2 - C3* | *C3 - C4* | **0.6** | **<.001** | **0.8** | **<.001** | **10.1** | **<.001** | **0.8** | **<.001** | **0.9** | **<.001** | **9.5** | **<.001** | **-1.0** | **<.001** | **1.3** | **<.001** |
|  | *C4 - C5* | **0.5** | **<.001** | **0.4** | **0.050** | **6.9** | **<.001** | **0.6** | **<.001** | **0.6** | **0.010** | **6.4** | **<.001** | **-1.6** | **<.001** | **1.2** | **<.001** |
|  | *C5 - C6* | **0.6** | **<.001** | -0.1 | 1.000 | **4.8** | **<.001** | **0.5** | **<.001** | -0.1 | 1.000 | **4.3** | **0.000** | **-2.0** | **<.001** | **0.7** | **<.001** |
|  | *C6 - C7* | **0.3** | **0.000** | -0.2 | 1.000 | **6.1** | **<.001** | **0.3** | **0.010** | -0.3 | 1.000 | **3.9** | **0.010** | **-1.2** | **<.001** | 0.2 | .115 |
|  | *C7 - T1* | **0.3** | **0.010** | -0.3 | 0.570 | **7.1** | **<.001** | **0.3** | **0.020** | -0.2 | 1.000 | **6.3** | **<.001** | * | * | **-0.6** | **<.001** |
| *C3 - C4* | *C2 - C3* | **-0.6** | **<.001** | **-0.8** | **<.001** | **-10.1** | **<.001** | **-0.8** | **<.001** | **-0.9** | **<.001** | **-9.5** | **<.001** | **1.0** | **<.001** | **-1.3** | **<.001** |
|  | *C4 - C5* | -0.1 | 1.000 | -0.4 | 0.120 | -3.2 | 0.060 | -0.2 | 0.370 | -0.3 | 1.000 | -3.1 | 0.120 | **-0.6** | **<.001** | -0.1 | .122 |
|  | *C5 - C6* | -0.1 | 1.000 | **-0.9** | **<.001** | **-5.3** | **<.001** | **-0.3** | **0.010** | **-1.0** | **<.001** | **-5.2** | **<.001** | **-1.0** | **<.001** | **-0.6** | **<.001** |
|  | *C6 - C7* | **-0.3** | **0.010** | **-1.0** | **<.001** | **-4.1** | **0.000** | **-0.5** | **<.001** | **-1.2** | **<.001** | **-5.6** | **<.001** | -0.3 | 0.196 | **-1.1** | **<.001** |
|  | *C7 - T1* | **-0.3** | **0.000** | **-1.1** | **<.001** | **-3.1** | **0.090** | **-0.5** | **<.001** | **-1.1** | **<.001** | -3.1 | 0.110 | * | * | **-1.9** | **<.001** |
| *C4 - C5* | *C2 - C3* | **-0.5** | **<.001** | **-0.4** | **0.050** | **-6.9** | **<.001** | **-0.6** | **<.001** | **-0.6** | **0.010** | **-6.4** | **<.001** | **1.6** | **<.001** | **-1.2** | **<.001** |
|  | *C3 - C4* | 0.1 | 1.000 | 0.4 | 0.120 | 3.2 | 0.060 | 0.2 | 0.370 | 0.3 | 1.000 | 3.1 | 0.120 | **0.6** | **<.001** | 0.1 | .122 |
|  | *C5 - C6* | 0.1 | 1.000 | **-0.5** | **0.010** | -2.1 | 0.950 | -0.1 | 1.000 | **-0.7** | **0.000** | -2.1 | 1.000 | **-0.4** | **0.006** | **-0.4** | **<.001** |
|  | *C6 - C7* | -0.2 | 0.730 | **-0.6** | **<.001** | -0.8 | 1.000 | **-0.3** | **0.010** | **-0.9** | **<.001** | -2.5 | 0.500 | **0.4** | **0.020** | **-1.0** | **<.001** |
|  | *C7 - T1* | -0.2 | 0.230 | **-0.7** | **<.001** | 0.2 | 1.000 | **-0.3** | **0.000** | **-0.8** | **<.001** | 0.0 | 1.000 | * | * | **-1.8** | **<.001** |
| *C5 - C6* | *C2 - C3* | **-0.6** | **<.001** | 0.1 | 1.000 | **-4.8** | **<.001** | **-0.5** | **<.001** | 0.1 | 1.000 | **-4.3** | **0.000** | **2.0** | **<.001** | **-0.7** | **<.001** |
|  | *C3 - C4* | 0.1 | 1.000 | **0.9** | **<.001** | **5.3** | **<.001** | **0.3** | **0.010** | **1.0** | **<.001** | **5.2** | **<.001** | **1.0** | **<.001** | **0.6** | **<.001** |
|  | *C4 - C5* | -0.1 | 1.000 | **0.5** | **0.010** | 2.1 | 0.950 | 0.1 | 1.000 | **0.7** | **0.000** | 2.1 | 1.000 | **0.4** | **0.006** | **0.4** | **<.001** |
|  | *C6 - C7* | -0.2 | 0.100 | -0.1 | 1.000 | 1.3 | 1.000 | -0.2 | 0.460 | -0.2 | 1.000 | -0.4 | 1.000 | **0.7** | **<.001** | **-0.6** | **<.001** |
|  | *C7 - T1* | **-0.3** | **0.020** | -0.2 | 1.000 | 2.2 | 0.680 | -0.2 | 0.200 | -0.1 | 1.000 | 2.0 | 1.000 | * | * | **-1.4** | **<.001** |
| *C6 - C7* | *C2 - C3* | **-0.3** | **0.000** | 0.2 | 1.000 | **-6.1** | **<.001** | **-0.3** | **0.010** | 0.3 | 1.000 | **-3.9** | **0.010** | **1.2** | **<.001** | -0.2 | 0.115 |
|  | *C3 - C4* | **0.3** | **0.010** | **1.0** | **<.001** | **4.1** | **0.000** | **0.5** | **<.001** | **1.2** | **<.001** | **5.6** | **<.001** | 0.3 | 0.196 | **1.1** | **<.001** |
|  | *C4 - C5* | 0.2 | 0.730 | **0.6** | **<.001** | 0.8 | 1.000 | **0.3** | **0.010** | **0.9** | **<.001** | 2.5 | 0.500 | **-0.4** | **0.020** | **1.0** | **<.001** |
|  | *C5 - C6* | 0.2 | 0.100 | 0.1 | 1.000 | -1.3 | 1.000 | 0.2 | 0.460 | 0.2 | 1.000 | 0.4 | 1.000 | **-0.7** | **<.001** | **0.6** | **<.001** |
|  | *C7 - T1* | 0.0 | 1.000 | -0.1 | 1.000 | 1.0 | 1.000 | 0.0 | 1.000 | 0.1 | 1.000 | 2.4 | 0.550 | * | * | **-0.8** | **<.001** |
| *C7 - T1* | *C2 - C3* | **-0.3** | **0.010** | 0.3 | 0.570 | **-7.1** | **<.001** | **-0.3** | **0.020** | 0.2 | 1.000 | **-6.3** | **<.001** | * | * | **0.6** | **<.001** |
|  | *C3 - C4* | **0.3** | **0.000** | **1.1** | **<.001** | 3.1 | 0.090 | **0.5** | **<.001** | **1.1** | **<.001** | 3.1 | 0.110 | * | * | **1.9** | **<.001** |
|  | *C4 - C5* | 0.2 | 0.230 | **0.7** | **<.001** | -0.2 | 1.000 | **0.3** | **0.000** | **0.8** | **<.001** | 0.0 | 1.000 | * | * | **1.8** | **<.001** |
|  | *C5 - C6* | **0.3** | **0.020** | 0.2 | 1.000 | -2.2 | 0.680 | 0.2 | 0.200 | 0.1 | 1.000 | -2.0 | 1.000 | * | * | **1.4** | **<.001** |
|  | *C6 - C7* | 0.0 | 1.000 | 0.1 | 1.000 | -1.0 | 1.000 | 0.0 | 1.000 | -0.1 | 1.000 | -2.4 | 0.550 | * | * | **0.8** | **<.001** |
