# Supplementary material for: Anatomic parameters for diagnosing congenital cervical stenosis via computed tomography
Source: Surg Radiol Anat. 2026 Jan 5;48(1):32. doi: 10.1007/s00276-025-03797-4 (PMC12769553; doi:10.1007/s00276-025-03797-4)
Supplement: Supplementary file 3 — Supplementary Material 3 [file 276_2025_3797_MOESM3_ESM.docx]

| **Supplementary Table 3. Differences in NFD, IPD, and APD Measurements based on Patient Sex** | | | | | | | | | | | |
| --- | --- | --- | --- | --- | --- | --- | --- | --- | --- | --- | --- |
| **Measurement** | | | **Male** | | | **Female** | | **Mean Difference (Male - Female)** | **95% Confidence Interval** | | ***p­-value*** |
|  |  |  | **Mean** | | **SD** | **Mean** | **SD** |  | **Lower Bound** | **Upper Bound** |  |
| *C2 - C3* | *Left NFD* | *Width* | 6.9 | 1.6 | | 7.2 | 1.6 | -0.3 | -0.5 | 0.0 | **0.021** |
|  |  | *Height* | 10.1 | 3.2 | | 8.8 | 1.7 | 1.3 | 0.9 | 1.7 | **<.001** |
|  |  | *Area* | 72.3 | 23.7 | | 57.8 | 19.7 | 14.5 | 11.2 | 17.7 | **<.001** |
|  | *Right NFD* | *Width* | 6.9 | 1.7 | | 7.3 | 1.5 | -0.3 | -0.6 | -0.1 | **0.010** |
|  |  | *Height* | 10.1 | 4.4 | | 8.9 | 1.7 | 1.2 | 0.7 | 1.7 | **<.001** |
|  |  | *Area* | 72.1 | 24.2 | | 58.3 | 20.5 | 13.8 | 10.5 | 17.1 | **<.001** |
|  | *IPD* | *C3* | 24.4 | 1.7 | | 23.8 | 1.2 | 0.6 | 0.3 | 0.9 | **<.001** |
|  | *APD* |  | 15.6 | 2.2 | | 15.4 | 2.2 | 0.2 | -1.1 | -0.5 | **<.001** |
| *C3 - C4* | *Left NFD* | *Width* | 6.2 | 1.6 | | 6.6 | 1.5 | -0.4 | -0.6 | -0.1 | **0.003** |
|  |  | *Height* | 9.1 | 2.9 | | 8.3 | 1.5 | 0.9 | 0.5 | 1.2 | **<.001** |
|  |  | *Area* | 59.1 | 18.9 | | 51.3 | 16.2 | 7.8 | 5.2 | 10.4 | **<.001** |
|  | *Right NFD* | *Width* | 6.1 | 1.6 | | 6.6 | 1.5 | -0.5 | -0.7 | -0.3 | **<.001** |
|  |  | *Height* | 9.1 | 1.7 | | 8.3 | 1.4 | 0.8 | 0.6 | 1.1 | **<.001** |
|  |  | *Area* | 60.3 | 19.6 | | 51.4 | 17.5 | 9.0 | 6.2 | 11.8 | **<.001** |
|  | *IPD* | *C4* | 25.4 | 1.7 | | 24.7 | 1.5 | 0.6 | 0.3 | 1.0 | **<.001** |
|  | *APD* |  | 14.3 | 1.7 | | 14.0 | 1.7 | 0.3 | -0.7 | -0.2 | **<.001** |
| *C4 - C5* | *Left NFD* | *Width* | 6.4 | 1.4 | | 6.7 | 1.5 | -0.3 | -0.5 | -0.1 | **0.006** |
|  |  | *Height* | 9.4 | 1.8 | | 8.8 | 1.6 | 0.7 | 0.4 | 0.9 | **<.001** |
|  |  | *Area* | 62.9 | 19.5 | | 53.9 | 16.8 | 9.0 | 6.3 | 11.7 | **<.001** |
|  | *Right NFD* | *Width* | 6.4 | 1.4 | | 6.6 | 1.4 | -0.3 | -0.5 | -0.1 | **0.012** |
|  |  | *Height* | 9.3 | 1.7 | | 8.8 | 4.4 | 0.4 | 0.0 | 0.9 | 0.070 |
|  |  | *Area* | 63.8 | 20.2 | | 53.8 | 17.0 | 9.9 | 7.1 | 12.8 | **<.001** |
|  | *IPD* | *C5* | 26.1 | 1.9 | | 25.2 | 1.6 | 0.9 | 0.6 | 1.2 | **<.001** |
|  | *APD* |  | 14.5 | 1.6 | | 14.1 | 1.6 | 0.4 | -0.7 | -0.2 | **<.001** |
| *C5 - C6* | *Left NFD* | *Width* | 6.3 | 1.5 | | 6.6 | 1.3 | -0.3 | -0.5 | -0.1 | **0.003** |
|  |  | *Height* | 9.9 | 4.1 | | 9.1 | 1.6 | 0.7 | 0.3 | 1.2 | **0.002** |
|  |  | *Area* | 63.8 | 19.1 | | 56.7 | 16.5 | 7.2 | 4.5 | 9.9 | **<.001** |
|  | *Right NFD* | *Width* | 6.3 | 1.4 | | 6.9 | 1.4 | -0.6 | -0.8 | -0.3 | **<.001** |
|  |  | *Height* | 9.6 | 1.9 | | 9.5 | 6.9 | 0.1 | -0.6 | 0.8 | 0.790 |
|  |  | *Area* | 64.5 | 20.1 | | 57.6 | 18.8 | 6.9 | 4.0 | 9.8 | **<.001** |
|  | *IPD* | *C6* | 26.5 | 1.9 | | 25.7 | 1.6 | 0.8 | 0.5 | 1.1 | **<.001** |
|  | *APD* |  | 15.0 | 1.9 | | 14.3 | 1.8 | 0.7 | -1.0 | -0.5 | **<.001** |
| *C6 - C7* | *Left NFD* | *Width* | 6.5 | 1.3 | | 6.8 | 1.5 | -0.2 | -0.4 | 0.0 | **0.024** |
|  |  | *Height* | 10.0 | 2.0 | | 9.3 | 1.8 | 0.6 | 0.3 | 0.9 | **<.001** |
|  |  | *Area* | 62.5 | 18.0 | | 56.2 | 16.5 | 6.4 | 3.8 | 8.9 | **<.001** |
|  | *Right NFD* | *Width* | 6.6 | 1.4 | | 7.0 | 1.5 | -0.4 | -0.6 | -0.2 | **<.001** |
|  |  | *Height* | 10.0 | 2.0 | | 9.5 | 3.7 | 0.5 | 0.0 | 0.9 | **0.033** |
|  |  | *Area* | 65.4 | 21.2 | | 57.3 | 19.0 | 8.1 | 5.1 | 11.2 | **<.001** |
|  | *IPD* | *C7* | 25.7 | 1.9 | | 25.0 | 1.6 | 0.7 | 0.4 | 1.1 | **<.001** |
|  | *APD* |  | 15.8 | 2.2 | | 14.9 | 2.1 | 0.9 | -1.5 | -0.9 | **<.001** |
| *C7 - T1* | *Left NFD* | *Width* | 6.8 | 1.4 | | 6.6 | 1.4 | 0.2 | 0.0 | 0.4 | **0.028** |
|  |  | *Height* | 10.2 | 2.0 | | 9.2 | 1.9 | 1.0 | 0.7 | 1.3 | **<.001** |
|  |  | *Area* | 63.5 | 19.6 | | 52.9 | 17.1 | 10.6 | 7.8 | 13.3 | **<.001** |
|  | *Right NFD* | *Width* | 6.8 | 1.4 | | 6.7 | 1.4 | 0.1 | -0.1 | 0.3 | 0.503 |
|  |  | *Height* | 10.2 | 2.0 | | 9.1 | 1.7 | 1.0 | 0.8 | 1.3 | **<.001** |
|  |  | *Area* | 64.5 | 19.9 | | 52.9 | 16.9 | 11.6 | 8.9 | 14.4 | **<.001** |
|  | *APD* |  | 16.7 | 2.3 | | 15.6 | 2.3 | 1.1 | -1.7 | -1.2 | **<.001** |
