# Supplementary material for: Anatomic parameters for diagnosing congenital cervical stenosis via computed tomography
Source: Surg Radiol Anat. 2026 Jan 5;48(1):32. doi: 10.1007/s00276-025-03797-4 (PMC12769553; doi:10.1007/s00276-025-03797-4)
Supplement: Supplementary file 4 — Supplementary Material 4 [file 276_2025_3797_MOESM4_ESM.docx]

| **Supplementary Table 4. Mean NFD, IPD, and APD Measurements based on Patient Race and Ethnicity** | | | | | | | | | | | | |
| --- | --- | --- | --- | --- | --- | --- | --- | --- | --- | --- | --- | --- |
| **Measurement** | | | **African American** | | **White** | | **Hispanic** | | **Asian** | | **Other** | |
|  |  |  | **Mean** | **SD** | **Mean** | **SD** | **Mean** | **SD** | **Mean** | **SD** | **Mean** | **SD** |
| *C2 - C3* | *Left NFD* | *Width* | 6.8 | 2.0 | 7.1 | 1.5 | 7.0 | 1.5 | 7.1 | 1.5 | 7.6 | 1.8 |
|  |  | *Height* | 9.3 | 1.8 | 9.9 | 1.9 | 9.5 | 3.3 | 9.3 | 1.8 | 9.4 | 1.5 |
|  |  | *Area* | 63.1 | 20.6 | 70.5 | 21.8 | 64.9 | 24.1 | 62.6 | 25.5 | 59.0 | 22.4 |
|  | *Right NFD* | *Width* | 6.8 | 1.6 | 7.1 | 1.5 | 7.1 | 1.6 | 7.4 | 1.9 | 7.8 | 1.8 |
|  |  | *Height* | 9.2 | 1.9 | 9.8 | 1.9 | 9.6 | 4.5 | 9.3 | 2.1 | 9.1 | 1.5 |
|  |  | *Area* | 61.1 | 20.9 | 70.7 | 21.9 | 65.8 | 24.1 | 59.1 | 27.6 | 61.7 | 27.9 |
|  | *IPD* | *C3* | 24.0 | 1.6 | 24.6 | 1.7 | 24.0 | 1.5 | 24.2 | 1.6 | 24.5 | 1.2 |
|  | *APD* |  | 14.6 | 2.1 | 15.4 | 2.1 | 15.2 | 2.3 | 14.7 | 2.2 | 15.1 | 1.7 |
| *C3 - C4* | *Left NFD* | *Width* | 6.3 | 1.8 | 6.4 | 1.5 | 6.4 | 1.6 | 6.6 | 1.3 | 7.0 | 1.3 |
|  |  | *Height* | 8.4 | 1.7 | 9.1 | 3.7 | 8.7 | 1.7 | 8.7 | 1.5 | 8.8 | 2.1 |
|  |  | *Area* | 51.5 | 16.1 | 58.7 | 16.5 | 55.9 | 19.1 | 54.2 | 18.4 | 51.1 | 17.7 |
|  | *Right NFD* | *Width* | 6.2 | 1.6 | 6.3 | 1.6 | 6.4 | 1.5 | 6.3 | 1.7 | 6.6 | 2.2 |
|  |  | *Height* | 8.6 | 1.7 | 8.9 | 1.6 | 8.7 | 1.7 | 8.7 | 1.5 | 8.3 | 1.4 |
|  |  | *Area* | 52.3 | 17.2 | 59.4 | 18.4 | 56.9 | 19.5 | 52.5 | 23.7 | 46.1 | 18.7 |
|  | *IPD* | *C4* | 25.0 | 1.4 | 25.5 | 1.8 | 25.0 | 1.6 | 24.8 | 1.8 | 25.5 | 1.1 |
|  | *APD* |  | 13.7 | 1.8 | 14.3 | 1.8 | 14.1 | 1.6 | 13.8 | 2.1 | 14.7 | 2.0 |
| *C4 - C5* | *Left NFD* | *Width* | 6.4 | 1.6 | 6.6 | 1.6 | 6.5 | 1.4 | 6.5 | 1.6 | 6.7 | 1.5 |
|  |  | *Height* | 8.7 | 1.7 | 9.4 | 1.8 | 9.1 | 1.7 | 9.0 | 1.9 | 9.5 | 2.1 |
|  |  | *Area* | 54.5 | 17.9 | 61.8 | 18.9 | 59.1 | 18.9 | 53.4 | 18.6 | 58.1 | 18.4 |
|  | *Right NFD* | *Width* | 6.3 | 1.4 | 6.4 | 1.4 | 6.5 | 1.4 | 6.7 | 1.5 | 6.5 | 1.7 |
|  |  | *Height* | 8.7 | 1.5 | 9.6 | 5.8 | 9.0 | 1.7 | 8.6 | 1.7 | 8.7 | 1.3 |
|  |  | *Area* | 57.0 | 17.3 | 62.8 | 18.3 | 59.2 | 20.0 | 54.3 | 27.6 | 53.0 | 18.0 |
|  | *IPD* | *C5* | 25.7 | 1.7 | 26.2 | 2.0 | 25.6 | 1.8 | 25.7 | 1.7 | 26.1 | 1.3 |
|  | *APD* |  | 13.9 | 1.7 | 14.5 | 1.7 | 14.2 | 1.6 | 14.0 | 2.0 | 15.3 | 1.7 |
| *C5 - C6* | *Left NFD* | *Width* | 6.4 | 1.5 | 6.3 | 1.4 | 6.5 | 1.4 | 6.3 | 0.8 | 7.1 | 1.5 |
|  |  | *Height* | 9.0 | 1.6 | 9.6 | 1.7 | 9.7 | 4.1 | 9.2 | 1.6 | 9.5 | 1.7 |
|  |  | *Area* | 57.3 | 15.6 | 63.4 | 17.9 | 61.0 | 19.0 | 50.9 | 14.2 | 58.8 | 21.8 |
|  | *Right NFD* | *Width* | 6.7 | 1.6 | 6.4 | 1.4 | 6.6 | 1.4 | 7.0 | 1.5 | 7.0 | 1.3 |
|  |  | *Height* | 9.9 | 8.9 | 9.9 | 6.2 | 9.4 | 2.0 | 9.1 | 2.1 | 9.3 | 1.5 |
|  |  | *Area* | 59.2 | 16.8 | 64.4 | 18.6 | 61.3 | 20.3 | 56.5 | 31.4 | 57.5 | 18.3 |
|  | *IPD* | *C6* | 26.1 | 1.8 | 26.6 | 1.8 | 1.9 | 0.9 | 26.3 | 2.6 | 25.9 | 1.3 |
|  | *APD* |  | 13.9 | 2.2 | 14.9 | 1.9 | 14.8 | 1.8 | 14.1 | 2.2 | 15.1 | 1.6 |
| *C6 - C7* | *Left NFD* | *Width* | 6.7 | 1.7 | 6.6 | 1.2 | 6.7 | 1.4 | 6.2 | 1.4 | 7.3 | 1.1 |
|  |  | *Height* | 9.3 | 1.7 | 9.8 | 1.7 | 9.8 | 2.0 | 8.9 | 1.8 | 9.5 | 2.0 |
|  |  | *Area* | 56.2 | 15.4 | 63.2 | 16.8 | 59.9 | 18.0 | 50.8 | 15.1 | 56.0 | 23.0 |
|  | *Right NFD* | *Width* | 6.8 | 1.7 | 6.6 | 1.3 | 6.8 | 1.4 | 6.9 | 1.8 | 7.3 | 1.7 |
|  |  | *Height* | 9.4 | 1.9 | 10.0 | 1.9 | 9.9 | 3.5 | 9.4 | 2.1 | 9.7 | 2.1 |
|  |  | *Area* | 58.3 | 16.7 | 65.8 | 20.7 | 61.7 | 20.8 | 56.3 | 31.3 | 56.6 | 19.5 |
|  | *IPD* | *C7* | 25.3 | 1.8 | 25.8 | 1.9 | 25.2 | 1.8 | 25.4 | 1.9 | 25.6 | 1.5 |
|  | *APD* |  | 14.5 | 2.0 | 15.6 | 2.4 | 15.3 | 2.2 | 14.6 | 2.3 | 15.2 | 2.2 |
| *C7 - T1* | *Left NFD* | *Width* | 6.5 | 1.5 | 6.8 | 1.4 | 6.7 | 1.4 | 6.5 | 1.5 | 6.5 | 1.1 |
|  |  | *Height* | 9.4 | 1.8 | 10.2 | 2.0 | 9.8 | 2.1 | 9.1 | 1.9 | 9.3 | 1.9 |
|  |  | *Area* | 53.1 | 16.4 | 64.1 | 19.4 | 58.4 | 19.0 | 52.1 | 20.0 | 53.0 | 21.9 |
|  | *Right NFD* | *Width* | 6.7 | 1.6 | 6.8 | 1.5 | 6.8 | 1.3 | 6.5 | 1.1 | 7.0 | 1.2 |
|  |  | *Height* | 9.3 | 1.4 | 9.9 | 1.9 | 9.8 | 2.0 | 9.4 | 2.2 | 9.4 | 1.6 |
|  |  | *Area* | 54.6 | 15.7 | 62.8 | 19.2 | 59.6 | 19.9 | 57.5 | 28.1 | 51.3 | 15.5 |
|  | *APD* |  | 15.4 | 2.1 | 16.4 | 2.4 | 16.1 | 2.4 | 15.6 | 2.1 | 16.2 | 2.0 |
