# Supplementary material for: Anatomic parameters for diagnosing congenital cervical stenosis via computed tomography
Source: Surg Radiol Anat. 2026 Jan 5;48(1):32. doi: 10.1007/s00276-025-03797-4 (PMC12769553; doi:10.1007/s00276-025-03797-4)
Supplement: Supplementary file 5 — Supplementary Material 5 [file 276_2025_3797_MOESM5_ESM.docx]

| **Supplementary Table 5. Mean Differences in NFD, IPD, and APD Measurements among African American, White, Hispanic, and Asian Cohorts** | | | | | | | | | | | | | | | | | | |
| --- | --- | --- | --- | --- | --- | --- | --- | --- | --- | --- | --- | --- | --- | --- | --- | --- | --- | --- |
| **Disc Level** | **Reference** | **Comparison** | **Mean Difference (Reference - Comparison)** | | | | | | | | | | | | | | | |
|  |  |  | **Left NFD Width** | | **Left NFD Height** | | **Left NFD Area** | | **Right NFD Width** | | **Right NFD Height** | | **Right NFD Area** | | **IPD** | | **APD** | |
|  |  |  | **MD** | ***p*** | **MD** | ***p*** | **MD** | ***p*** | **MD** | ***p*** | **MD** | ***p*** | **MD** | ***p*** | **MD** | ***p*** | **MD** | ***p*** |
| *C2 – C3* | *African American* | *White* | -0.3 | 0.542 | -0.6 | 0.297 | **-7.4** | **0.029** | -0.3 | 0.31 | -0.6 | 0.556 | **-9.6** | **0.002** | **-0.6** | **0.034** | **-0.8** | **0.008** |
|  | *African American* | *Hispanic* | -0.2 | 0.809 | -0.2 | 1 | -1.8 | 1 | -0.3 | 0.272 | -0.4 | 0.996 | -4.7 | 0.199 | -0.1 | 1 | **-0.6** | **0.049** |
|  | *African American* | *Asian* | -0.3 | 1 | 0.1 | 1 | 0.5 | 1 | -0.6 | 1 | -0.1 | 1 | 1.9 | 1 | -0.2 | 1 | -0.06 | 1 |
|  | *White* | *African American* | 0.3 | 0.542 | 0.6 | 0.297 | **7.4** | **0.029** | 0.3 | 0.31 | 0.6 | 0.556 | **9.6** | **0.002** | **0.6** | **0.034** | **0.8** | **0.008** |
|  | *White* | *Hispanic* | 0.1 | 1 | 0.4 | 0.32 | **5.6** | **0.021** | 0 | 1 | 0.2 | 1 | 4.9 | 0.056 | **0.5** | **0.008** | 0.2 | 0.755 |
|  | *White* | *Asian* | 0.0 | 1 | 0.6 | 1 | 7.9 | 1 | -0.2 | 1 | 0.5 | 1 | 11.6 | 0.398 | 0.4 | 1 | 0.8 | 0.48 |
|  | *Hispanic* | *African American* | 0.2 | 0.809 | 0.2 | 1 | 1.8 | 1 | 0.3 | 0.272 | 0.4 | 0.996 | 4.7 | 0.199 | 0.1 | 1 | **0.6** | **0.049** |
|  | *Hispanic* | *White* | -0.1 | 1 | -0.4 | 0.32 | **-5.6** | **0.021** | 0 | 1 | -0.2 | 1 | -4.9 | 0.056 | **-0.5** | **0.008** | -0.2 | 0.755 |
|  | *Hispanic* | *Asian* | -0.1 | 1 | 0.2 | 1 | 2.3 | 1 | -0.3 | 1 | 0.3 | 1 | 6.7 | 1 | -0.1 | 1 | 0.6 | 0.74 |
|  | *Asian* | *African American* | 0.3 | 1 | -0.1 | 1.0 | -0.5 | 1.0 | 0.6 | 1.0 | 0.1 | 1.0 | -1.9 | 1.0 | 0.2 | 1 | 0.06 | 1 |
|  | *Asian* | *White* | 0.0 | 1 | -0.6 | 1.0 | -7.9 | 1.0 | 0.2 | 1.0 | -0.5 | 1.0 | -11.6 | 0.4 | -0.4 | 1 | -0.8 | 0.48 |
|  | *Asian* | *Hispanic* | 0.1 | 1 | -0.2 | 1.0 | -2.3 | 1.0 | 0.3 | 1.0 | -0.3 | 1.0 | -6.7 | 1.0 | 0.1 | 1 | -0.6 | 0.74 |
| *C3 - C4* | *African American* | *White* | -0.1 | 1 | **-0.8** | **0.036** | **-7.2** | **0.004** | -0.1 | 1 | -0.3 | 0.391 | **-7** | **0.008** | -0.5 | 0.158 | **-0.67** | **0.005** |
|  | *African American* | *Hispanic* | -0.1 | 1 | -0.3 | 0.694 | -4.4 | 0.092 | -0.2 | 1 | -0.1 | 1 | -4.6 | 0.087 | 0.1 | 1 | -0.44 | 0.093 |
|  | *African American* | *Asian* | -0.3 | 1 | -0.3 | 1.0 | -2.7 | 1.0 | -0.1 | 1.0 | -0.1 | 1.0 | -0.2 | 1.0 | 0.2 | 1 | -0.18 | 0.990 |
|  | *White* | *African American* | 0.1 | 1 | **0.8** | **0.036** | **7.2** | **0.004** | 0.1 | 1 | 0.3 | 0.391 | **7** | **0.008** | 0.5 | 0.158 | **0.67** | **0.005** |
|  | *White* | *Hispanic* | 0 | 1 | 0.4 | 0.147 | 2.8 | 0.255 | -0.1 | 1 | 0.2 | 0.811 | 2.4 | 0.464 | **0.6** | **0.005** | 0.23 | 0.461 |
|  | *White* | *Asian* | -0.2 | 1 | 0.4 | 1.0 | 4.5 | 1.0 | 0.0 | 1.0 | 0.2 | 1.0 | 6.9 | 1.0 | 0.7 | 1 | 0.49 | 0.679 |
|  | *Hispanic* | *African American* | 0.1 | 1 | 0.3 | 0.694 | 4.4 | 0.092 | 0.2 | 1 | 0.1 | 1 | 4.6 | 0.087 | -0.1 | 1 | 0.44 | 0.093 |
|  | *Hispanic* | *White* | 0 | 1 | -0.4 | 0.147 | -2.8 | 0.255 | 0.1 | 1 | -0.2 | 0.811 | -2.4 | 0.464 | **-0.6** | **0.005** | -0.23 | 0.461 |
|  | *Hispanic* | *Asian* | -0.2 | 1 | 0.0 | 1.0 | 1.7 | 1.0 | 0.0 | 1.0 | 0.1 | 1.0 | 4.4 | 1.0 | 0.1 | 1 | 0.26 | 0.952 |
|  | *Asian* | *African American* | 0.3 | 1 | 0.3 | 1.0 | 2.7 | 1.0 | 0.1 | 1.0 | 0.1 | 1.0 | 0.2 | 1.0 | -0.2 | 1 | 0.18 | 0.990 |
|  | *Asian* | *White* | 0.2 | 1 | -0.4 | 1.0 | -4.5 | 1.0 | 0.0 | 1.0 | -0.2 | 1.0 | -6.9 | 1.0 | -0.7 | 1 | -0.49 | 0.679 |
|  | *Asian* | *Hispanic* | 0.2 | 1 | 0.0 | 1.0 | -1.7 | 1.0 | 0.0 | 1.0 | -0.1 | 1.0 | -4.4 | 1.0 | -0.1 | 1 | -0.26 | 0.952 |
| *C4 - C5* | *African American* | *White* | -0.2 | 0.629 | **-0.7** | **0.001** | **-7.3** | **0.005** | -0.1 | 1 | -0.9 | 0.084 | **-5.7** | **0.049** | -0.5 | 0.246 | **-0.58** | **0.021** |
|  | *African American* | *Hispanic* | -0.2 | 1 | -0.4 | 0.082 | -4.6 | 0.087 | -0.2 | 0.549 | -0.3 | 1 | -2.2 | 0.895 | 0.1 | 1 | -0.30 | 0.423 |
|  | *African American* | *Asian* | -0.2 | 1 | -0.4 | 1.0 | 1.1 | 1.0 | -0.4 | 1.0 | 0.1 | 1.0 | 2.7 | 1.0 | -0.1 | 1 | -0.07 | 1 |
|  | *White* | *African American* | 0.2 | 0.629 | **0.7** | **0.001** | **7.3** | **0.005** | 0.1 | 1 | 0.9 | 0.084 | **5.7** | **0.049** | 0.5 | 0.246 | **0.58** | **0.021** |
|  | *White* | *Hispanic* | 0.1 | 1 | 0.3 | 0.105 | 2.7 | 0.322 | -0.1 | 0.965 | 0.6 | 0.133 | 3.5 | 0.132 | **0.6** | **0.01** | 0.28 | 0.231 |
|  | *White* | *Asian* | 0.0 | 1 | 0.4 | 1.0 | 8.4 | 0.6 | -0.3 | 1.0 | 1.1 | 1.0 | 8.4 | 0.8 | 0.4 | 1 | 0.51 | 0.623 |
|  | *Hispanic* | *African American* | 0.2 | 1 | 0.4 | 0.082 | 4.6 | 0.087 | 0.2 | 0.549 | 0.3 | 1 | 2.2 | 0.895 | -0.1 | 1 | 0.30 | 0.423 |
|  | *Hispanic* | *White* | -0.1 | 1 | -0.3 | 0.105 | -2.7 | 0.322 | 0.1 | 0.965 | -0.6 | 0.133 | -3.5 | 0.132 | **-0.6** | **0.01** | 0.28 | 0.231 |
|  | *Hispanic* | *Asian* | 0.0 | 1 | 0.1 | 1.0 | 5.6 | 1.0 | -0.2 | 1.0 | 0.4 | 1.0 | 4.9 | 1.0 | -0.2 | 1 | 0.23 | 0.967 |
|  | *Asian* | *African American* | 0.2 | 1 | 0.4 | 1.0 | -1.1 | 1.0 | 0.4 | 1.0 | -0.1 | 1.0 | -2.7 | 1.0 | 0.1 | 1 | -0.07 | 1 |
|  | *Asian* | *White* | 0.0 | 1 | -0.4 | 1.0 | -8.4 | 0.6 | 0.3 | 1.0 | -1.1 | 1.0 | -8.4 | 0.8 | -0.4 | 1 | 0.51 | 0.623 |
|  | *Asian* | *Hispanic* | 0.0 | 1 | -0.1 | 1.0 | -5.6 | 1.0 | 0.2 | 1.0 | -0.4 | 1.0 | -4.9 | 1.0 | 0.2 | 1 | -0.23 | 0.967 |
| *C5 - C6* | *African American* | *White* | 0.1 | 1 | -0.6 | 0.455 | **-6** | **0.023** | 0.2 | 0.521 | -0.1 | 1 | -5.2 | 0.092 | -0.5 | 0.221 | **-1.01** | **<0.001** |
|  | *African American* | *Hispanic* | -0.1 | 1 | -0.7 | 0.211 | -3.7 | 0.204 | 0.1 | 1 | 0.5 | 1 | -2 | 1 | 0.1 | 1 | **-0.85** | **<0.001** |
|  | *African American* | *Asian* | 0.1 | 1 | -0.2 | 1.0 | 6.4 | 1.0 | -0.3 | 1.0 | 0.8 | 1.0 | 2.8 | 1.0 | -0.2 | 1 | -0.14 | 0.998 |
|  | *White* | *African American* | -0.1 | 1 | 0.6 | 0.455 | **6** | **0.023** | -0.2 | 0.521 | 0.1 | 1 | 5.2 | 0.092 | 0.5 | 0.221 | **1.01** | **<0.001** |
|  | *White* | *Hispanic* | -0.2 | 0.503 | -0.1 | 1 | 2.3 | 0.47 | -0.2 | 0.734 | 0.5 | 0.75 | 3.2 | 0.213 | **0.6** | **0.004** | 0.15 | 0.855 |
|  | *White* | *Asian* | 0.0 | 1 | 0.4 | 1.0 | 12.5 | 0.0 | -0.6 | 1.0 | 0.9 | 1.0 | 8.0 | 1.0 | 0.3 | 1 | 0.87 | 0.215 |
|  | *Hispanic* | *African American* | 0.1 | 1 | 0.7 | 0.211 | 3.7 | 0.204 | -0.1 | 1 | -0.5 | 1 | 2 | 1 | -0.1 | 1 | **0.85** | **<0.001** |
|  | *Hispanic* | *White* | 0.2 | 0.503 | 0.1 | 1 | -2.3 | 0.47 | 0.2 | 0.734 | -0.5 | 0.75 | -3.2 | 0.213 | **-0.6** | **0.004** | -0.15 | 0.855 |
|  | *Hispanic* | *Asian* | 0.2 | 1 | 0.5 | 1.0 | 10.1 | 0.2 | -0.4 | 1.0 | 0.4 | 1.0 | 4.8 | 1.0 | -0.4 | 1 | 0.71 | 0.384 |
|  | *Asian* | *African American* | -0.1 | 1 | 0.2 | 1.0 | -6.4 | 1.0 | 0.3 | 1.0 | -0.8 | 1.0 | -2.8 | 1.0 | 0.2 | 1 | 0.14 | 0.998 |
|  | *Asian* | *White* | 0.0 | 1 | -0.4 | 1.0 | -12.5 | 0.0 | 0.6 | 1.0 | -0.9 | 1.0 | -8.0 | 1.0 | -0.3 | 1 | -0.87 | 0.215 |
|  | *Asian* | *Hispanic* | 0.0 | 1 | -0.5 | 1.0 | -10.1 | 0.2 | 0.4 | 1.0 | -0.4 | 1.0 | -4.8 | 1.0 | 0.4 | 1 | -0.71 | 0.384 |
| *C6 - C7* | *African American* | *White* | 0.1 | 1 | -0.5 | 0.116 | **-7** | **0.004** | 0.2 | 0.944 | -0.5 | 0.439 | **-7.5** | **0.009** | -0.5 | 0.208 | **-1.01** | **0.001** |
|  | *African American* | *Hispanic* | 0 | 1 | -0.5 | 0.104 | -3.7 | 0.175 | 0 | 1 | -0.4 | 0.605 | -3.4 | 0.407 | 0.1 | 1 | -0.70 | 0.022 |
|  | *African American* | *Asian* | 0.5 | 1 | 0.5 | 1.0 | 5.4 | 1.0 | -0.1 | 1.0 | 0.1 | 1.0 | 2.1 | 1.0 | -0.1 | 1 | 0.05 | 1 |
|  | *White* | *African American* | -0.1 | 1 | 0.5 | 0.116 | **7** | **0.004** | -0.2 | 0.944 | 0.5 | 0.439 | **7.5** | **0.009** | 0.5 | 0.208 | **1.01** | **0.001** |
|  | *White* | *Hispanic* | -0.1 | 1 | 0 | 1 | 3.3 | 0.106 | -0.1 | 0.716 | 0.1 | 1 | 4.1 | 0.077 | **0.5** | **0.017** | 0.31 | 0.443 |
|  | *White* | *Asian* | 0.4 | 1 | 0.9 | 0.4 | 12.3 | 0.0 | -0.3 | 1.0 | 0.6 | 1.0 | 9.5 | 0.6 | 0.4 | 1 | 1.05 | 0.191 |
|  | *Hispanic* | *African American* | 0 | 1 | 0.5 | 0.104 | 3.7 | 0.175 | 0 | 1 | 0.4 | 0.605 | 3.4 | 0.407 | -0.1 | 1 | 0.70 | 0.022 |
|  | *Hispanic* | *White* | 0.1 | 1 | 0 | 1 | -3.3 | 0.106 | 0.1 | 0.716 | -0.1 | 1 | -4.1 | 0.077 | **-0.5** | **0.017** | -0.31 | 0.443 |
|  | *Hispanic* | *Asian* | 0.5 | 1 | 0.9 | 0.4 | 9.0 | 0.3 | -0.1 | 1.0 | 0.5 | 1.0 | 5.4 | 1.0 | -0.1 | 1 | 0.75 | 0.509 |
|  | *Asian* | *African American* | -0.5 | 1 | -0.5 | 1.0 | -5.4 | 1.0 | 0.1 | 1.0 | -0.1 | 1.0 | -2.1 | 1.0 | 0.1 | 1 | -0.046 | 1 |
|  | *Asian* | *White* | -0.4 | 1 | -0.9 | 0.4 | -12.3 | 0.0 | 0.3 | 1.0 | -0.6 | 1.0 | -9.5 | 0.6 | -0.4 | 1 | -1.05 | 0.191 |
|  | *Asian* | *Hispanic* | -0.5 | 1 | -0.9 | 0.4 | -9.0 | 0.3 | 0.1 | 1.0 | -0.5 | 1.0 | -5.4 | 1.0 | 0.1 | 1 | -0.75 | 0.509 |
| *C7 - T1* | *African American* | *White* | -0.3 | 0.209 | **-0.8** | **0.005** | **-11** | **<.001** | -0.1 | 1 | **-0.6** | **0.022** | **-8.2** | **0.002** | * | * | **-1.02** | **0.002** |
|  | *African American* | *Hispanic* | -0.2 | 0.444 | -0.4 | 0.205 | **-5.3** | **0.033** | -0.1 | 1 | **-0.5** | **0.041** | -5 | 0.057 | * | * | -0.69 | 0.042 |
|  | *African American* | *Asian* | 0.0 | 1 | 0.2 | 1.0 | 1.0 | 1.0 | 0.2 | 1.0 | -0.2 | 1.0 | -2.9 | 1.0 | * | * | -0.16 | 0.998 |
|  | *White* | *African American* | 0.3 | 0.209 | **0.8** | **0.005** | **11** | **<.001** | 0.1 | 1 | **0.6** | **0.022** | **8.2** | **0.002** | * | * | **1.02** | **0.002** |
|  | *White* | *Hispanic* | 0.1 | 1 | 0.4 | 0.114 | **5.7** | **0.002** | 0 | 1 | 0.1 | 1 | 3.1 | 0.211 | * | * | 0.33 | 0.401 |
|  | *White* | *Asian* | 0.3 | 1 | 1.0 | 0.4 | 12.0 | 0.1 | 0.3 | 1.0 | 0.5 | 1.0 | 5.3 | 1.0 | * | * | 0.86 | 0.447 |
|  | *Hispanic* | *African American* | 0.2 | 0.444 | 0.4 | 0.205 | **5.3** | **0.033** | 0.1 | 1 | **0.5** | **0.041** | 5 | 0.057 | * | * | 0.69 | 0.042 |
|  | *Hispanic* | *White* | -0.1 | 1 | -0.4 | 0.114 | **-5.7** | **0.002** | 0 | 1 | -0.1 | 1 | -3.1 | 0.211 | * | * | -0.33 | 0.401 |
|  | *Hispanic* | *Asian* | 0.2 | 1 | 0.6 | 1.0 | 6.3 | 1.0 | 0.3 | 1.0 | 0.4 | 1.0 | 2.1 | 1.0 | * | * | 0.53 | 0.831 |
|  | *Asian* | *African American* | 0.0 | 1 | -0.2 | 1.0 | -1.0 | 1.0 | -0.2 | 1.0 | 0.2 | 1.0 | 2.9 | 1.0 | * | * | 0.16 | 0.998 |
|  | *Asian* | *White* | -0.3 | 1 | -1.0 | 0.4 | -12.0 | 0.1 | -0.3 | 1.0 | -0.5 | 1.0 | -5.3 | 1.0 | * | * | -0.86 | 0.447 |
|  | *Asian* | *Hispanic* | -0.2 | 1 | -0.6 | 1.0 | -6.3 | 1.0 | -0.3 | 1.0 | -0.4 | 1.0 | -2.1 | 1.0 | * | * | -0.53 | 0.831 |
